# Examining Risk Factors for Suicidality in Adolescents and Adults Experiencing Their First Episode of Psychosis

**DOI:** 10.7759/cureus.43135

**Published:** 2023-08-08

**Authors:** Gurtej Gill, Sanobar Jaka, Garima Yadav, Tejasvi Kainth, Yarden Segal, Sushma Srinivas, Kaushal Shah, Hansini Kochhar, Sasidhar Gunturu

**Affiliations:** 1 Psychiatry, BronxCare Health System, Bronx, USA; 2 Section on Tobacco, Alcohol and Drug Use, Department of Population Health, New York University Grossman School of Medicine, New York, USA; 3 Research, Basaveshwara Medical College and Hospital, Chitradurga, IND; 4 Language Access and Internal Medicine, Winnipeg Regional Health Authority, Winnipeg, CAN; 5 Psychiatry, A.J. Institute of Medical Sciences and Research Centre, Mangalore, IND; 6 Psychiatry, Griffin Memorial Hospital, Norman, USA; 7 Clinical and Translational Research, Larkin Community Hospital, Miami, USA

**Keywords:** first-episode psychosis, suicidal behavior, adolescent and young adults, depressive disorders, duration of untreated psychosis, suicidality

## Abstract

This narrative review aimed to identify the risk factors associated with suicidality in adolescents and adults with first-episode psychosis. The review included studies that examined various factors such as psychiatric, familial, and social factors, as well as previous self-harm, suicidal ideation, and comorbid mental health disorders. A comprehensive literature search was conducted across three publicly available databases (Embase, American Psychological Association PsycINFO, and PubMed) using specific search terms related to first-episode psychosis, suicide, self-harm, and children/adolescents and adults. The inclusion criteria included original articles focusing on prospective and retrospective cohort trials, with substantial data on first-episode psychosis and self-harm, measuring both suicidal intent and outcome. Non-original studies, case reports, case series, non-English-language publications, and studies examining violence and self-harm related to substance-induced psychosis were excluded. After manual screening and removing duplicate articles, 13 articles met the established criteria for inclusion in this review. Included studies adhered to similar inclusion and exclusion criteria, had long-term follow-up, and assessed outcomes at least twice. The findings suggest that depressive symptoms, substance use disorders, previous self-harm or suicidal ideation, and longer duration of untreated psychosis are associated with an increased risk of suicidality. However, insights into psychosis and premorbid intellectual functioning did not show a direct association with suicidality.

## Introduction and background

Psychosis is a known significant risk factor for suicidal ideation, suicidal attempt, and suicidal death [1]. Based on statistics from the National Institutes of Health, the prevalence of psychotic disorders in the United States is between 0.25% and 0.64%, with an estimated 3% of individuals experiencing at least one psychotic episode during their lifetime [2]. First-episode psychosis (FEP) refers to the initial occurrence of psychosis in an individual’s life. It marks the first time a person experiences symptoms of psychosis [3]. Every year, roughly 100,000 adolescents and young adults experience their first episode of psychosis [2]. The risk of violence in individuals with psychosis is highest during their initial episodes, particularly among younger age groups before beginning treatment [4]. The likelihood of violent behavior and aggression during FEP is nearly 16% [5]. A large UK-based study discovered that self-harm is prevalent among individuals with FEP, with over 10% engaging in self-harm before receiving treatment [6].

Adolescent patients with psychosis have a significantly high risk of attempted suicide, ranging from 12.4% to 72%, and a mortality rate between 2.73% and 4.5% [7-11]. Suicidal thoughts are reported in approximately 48% of these patients, particularly among outpatients and during the first month of hospitalization [8,10]. Compared to healthy peers, patients with depression, and non-psychotic psychiatric patients, the risk of suicidality was higher in the adolescent psychosis group [12,13]. Suicidal ideation, which predicts suicide and guides prevention efforts, was experienced frequently by 40% of patients and persisted or increased during treatment [14,15]. The significance of these findings surpasses that observed in adult populations with psychosis, indicating a 25-fold higher suicide risk for young individuals with psychotic disorders in comparison to their counterparts in the general population [16]. The prevalence of suicide in FEP patients is estimated to be 1-3% [17,18]. Early onset of psychosis is particularly associated with an increased suicide risk [19]. Adolescents with FEP have an increased risk of suicidal behavior [13]. In addition, pediatric psychosis is associated with an increased risk for multiple suicide attempts. Strong risk factors identified include longer duration of untreated psychosis, attention-deficit/hyperactivity disorder (ADHD), and depressive symptoms [13].

The main objective of this study was to conduct a comprehensive review and synthesis of the available literature concerning suicide and suicide attempts among adults as well as adolescents who are experiencing their first episode of psychosis. Our analysis focused on prospective and retrospective cohort studies that investigated the risk factors associated with suicidality in individuals with FEP from different regions across the globe. In addition, we aimed to assess the risk factors that contribute to an increased risk of suicide in FEP and to identify risk factors that may be particularly more associated with adolescents and young adults.

## Review

Methodology

Search Terminology

We performed an extensive literature search utilizing three publicly available databases: Embase, American Psychological Association PsycINFO, and PubMed. The search terms employed were (first episode psychosis OR FEP OR first episode schizophrenia) AND (suicide OR suicidality OR self-harm) AND (children OR adolescents). Our search criteria encompassed the period from January 2000 to January 2023.

Inclusion Criteria

This review incorporated original articles that met specific inclusion criteria. These criteria included articles focusing on prospective cohort and retrospective cohort trials, involving patients or participants experiencing FEP, and examining both suicidal intent and outcome. Additionally, studies with long-term follow-up in longitudinal designs and assessing outcomes at a minimum of two different time points were considered. Retrospective studies that utilized large datasets consisting of chart reviews spanning a period of five to seven years were also included.

Exclusion Criteria

Non-original studies, such as literature reviews, systematic reviews, meta-analyses, letters to the editor, as well as case series and reports, were excluded. Publications in languages other than English were also excluded. Furthermore, studies that examined the incidence of violence and self-harm in relation to substance-induced psychosis were excluded from our analysis. A total of 2,100 articles were retrieved, screened manually, and assessed using MS Excel. After eliminating duplicate entries, we applied our pre-established exclusion and inclusion criteria, resulting in the inclusion of 13 articles in this review.

Results

By implementing our search strategy, we identified 13 extensive cohort studies comprising individuals with FEP, as shown in Table 1.

**Table 1:** Characteristics of studies: participants, demographics, and study design. FEP: first-episode psychosis; AESOP: Aetiology and Ethnicity in Schizophrenia and Other Psychoses; GAP: genetics and psychosis

Study	Participants	Age (years)	Mean age (years)	Design
Björkenstam et al. (2014) [20]	2,819 patients with FEP	15–30	22.6	Prospective cohort study
Moe et al. (2022) [21]	19,422 adolescents and young adults diagnosed with the onset of FEP	15–24	Not reported	Retrospective cohort study
Robinson et al. (2010) [22]	413 FEP patients	15–30	21.3	Prospective cohort study
Bornheimer et al. (2019) [23]	404 FEP patients	15–40	23.6	Secondary data analysis of prospective cohort study
Mitter et al. (2013) [24]	1,397 FEP patients	15–41	28.2	Prospective cohort study
Canal-Rivero et al. (2019) [25]	65 FEP patients	14–60	26.2	Prospective cohort study
Lopez-Morinigo et al. (2019) [26]	AESOP: 181 participants	16–65	30.5	Prospective cohort study
Lopez-Morinigo et al. (2019) [26]	GAP: 112 participants	18–65	29.4	Prospective cohort study
Barret et al. (2010) [27]	194 FEP patients	18–65	27	Prospective cohort study
Fedyszyn et al. (2011) [8]	607 FEP patients	15–24	19.2	Retrospective cohort study
Canal-Rivero et al. (2018) [28]	517 FEP patients	15–60	28.3	Prospective cohort study
Ayesa-Arriola et al. (2018) [29]	397 FEP patients	15–60	29.8	Prospective cohort study
Pelizza et al. (2020) [30]	134 FEP patients	12–54	29.5	Prospective cohort study
Austad et al. (2015) [31]	246 FEP patients	15–65	26.6	Prospective cohort study

The studies encompassed participants aged 12 to 65 years, with a predominant focus on adolescents and transitional-age youth. The primary outcome, for the majority of the identified studies, focused on suicide rate, suicide attempt, or suicide ideation. Therefore, for the purpose of this review, these outcomes were categorized as suicidal behaviors. Eleven studies were prospective trials with follow-up periods ranging from two to seven years. Geographically, these studies were conducted across all continents, including Europe, North America, parts of Asia, and Oceania. The most significant risk factors for suicide risk in these cohorts were assessed using scales such as the Calgary Depression Scale for Depression (CDSS), the Positive and Negative Syndrome Scale (PANSS), the Global Assessment of Functioning (GAF), and Global Cognitive Functioning (GCF), as shown in Table 2.

**Table 2:** Risk factors in first episodes of psychosis for suicidality. DSH: deliberate self-harm; DSM-IV: Diagnostic and Statistical Manual of Mental Disorders, Fourth Edition; SA: suicide attempt; SB: suicidal behavior; SI: suicidal ideation; SAPS: Scales for the Assessment of Positive Symptoms; SANS: Scales for the Assessment of Negative Symptoms; CDSS: Calgary Depression Scale for Depression; DAS: Disability Assessment Scale; GCF: Global Cognitive Functioning; FEP: first-episode psychosis; BPRS: Brief Psychiatric Rating Scale

Study	Risk factors examined	Main findings in the FEP cohort
Björkenstam et al. (2014) [20]	Psychiatric factors, familial factors, social factors, premorbid intellectual functional level, and other risk factors that included head injury and obstetrical complication	The highest suicide rates were found in depressive disorder with psychotic symptoms as well as in delusional disorder. The risk factors for suicide were self-harm, a conviction for violent crime, having a first-degree relative with a schizophrenia/bipolar diagnosis, and substance use disorder
Moe et al. (2022) [21]	Previous DSH, suicidal ideation, child abuse and neglect, comorbid medical and psychiatric diagnoses, and prior mental health care	DSH rates were higher than suicide. Suicide was associated with personality disorders, substance use disorders, other mental health disorders, and a history of DSH or suicidal ideation
Robinson et al. (2010) [22]	Sociodemographic and clinical features, DSM-IV, duration of prodromal symptoms in days, psychopathology scores, hopelessness, family history of suicide, previous self-arm	Predictors of SA were previous self-harm, suicidal tendencies, hopelessness, and being depressed for more than half of the follow-up period
Bornheimer et al. (2019) [23]	Severity of depression symptoms in schizophrenia including SI with CDSS score	SI was higher than SA. SI was high among untreated psychosis. The majority of patients reported experiencing hallucinations, delusions, and varying degrees of depression. Significant higher depression scores at baseline were reported for those who reported SI during the study
Mitter et al. (2013) [24]	The severity of psychopathology using the PANSS score and functioning by the Global Assessment of Functioning scale	Older patients with a longer duration of psychosis, more severe symptoms, and better functioning were at an increased risk of suicide
Canal-Rivero et al. (2019) [25]	The severity of psychopathology using the PANSS score and functioning by the Global Assessment of Functioning scale, and personality traits using the PAS	Theory of mind impairments with more severe symptoms during the first contact with mental health services for psychotic symptoms was associated with multiple SAs. Multiple suicide attempters committed their first SA after FEP earlier than single suicide attempters
Lopez-Morinigo et al. (2019) [26]	Premorbid, sociodemographic, and clinical variables most importantly first contact with services and duration of untreated psychosis, insight assessment, PANSS score for psychopathological symptoms, and CDSS scores.	No direct association of insight with SB. Previous SA and depression were the main predictors of SB
Barret et al. (2010) [27]	PANSS and CDSS scores, belief about psychosis	Depressive symptoms did not mediate between insight and suicidality or between beliefs and suicidality. A large portion of the sample was currently suicidal
Fedyszyn et al. (2011) [8]	Demographic, clinical, and treatment information associated with SA	The majority of SA occurred when patients were treated as outpatients and were in regular contact with the services. SA tended to be impulsive and triggered by interpersonal conflict or distress due to psychotic symptoms
Canal-Rivero et al. (2018) [28]	Sociodemographic, clinical, functional, and neurocognitive measures	GCF and severe depressive symptoms predicted SB. The history of SB had worse GCF and visual memory than those without such antecedents
Ayesa-Arriola et al. (2018) [29]	Demographic, age at onset of psychosis, duration of untreated psychosis, PAS scale, information on SB, insight assessment	No associations were found between baseline insight dimensions and time to SB. Insight in psychosis is a dynamic concept and the relationship between insight and suicide risk is dynamic
Pelizza et al. (2020) [30]	Psychopathological assessment BPRS, PANSS, and GAF scale	SI was positively correlated with symptoms of depression
Austad et al. (2015) [31]	Sociodemographic characteristics, clinical characteristics, level of suicidality during lifetime	High SB in FEP patients. Young age, depressive symptoms, and female gender at high risk. Lifetime suicide prevalence risk was also associated with a longer duration of untreated psychosis

The prevalence of suicidal behavior in each study is presented in Table 3.

**Table 3:** Prevalence of suicidal behavior across studies. FEP: first-episode psychosis; AESOP: Aetiology and Ethnicity in Schizophrenia and Other Psychoses (AESOP); GAP: genetics and psychosis; T3: 7.4 years after the index presentation

Study	Prevalence of suicidal behavior
Björkenstam et al. (2014) [20]	121 patients died by suicide. The overall suicide rate was 4.3% (95% confidence interval (CI) = 3.5–5.0) per 1,000 person-years
Moe et al. (2022) [21]	The suicide standardized mortality ratio (SMR) for the total cohort was 4.5 (95% CI = 2.9–6.8) compared to the same calendar year, age, and gender general population. The suicide SMR was higher for adolescents (SMR = 5.4, 95% CI = 3.2–8.6) than for young adults (SMR = 3.2, 95% CI = 1.3–6.6)
Robinson et al. (2010) [22]	One-fifth (n = 61; 21.6%) of the study sample of 282 subjects had made an SA by the T3 follow-up; 12 of these (19.7%) died as a result
Bornheimer et al. (2019) [23]	Throughout the entire study period, 26% of participants endorsed having suicidal ideation and 8.2% made a suicide attempt
Mitter et al. (2013) [24]	26 patients (1.9%) had completed suicides
Canal-Rivero et al. (2019) [25]	A total of 20 participants attempted suicide during the follow-up period. Fourteen (70%) suicide attempts occurred in the following six months after FEP. Five (35.71%) of the first suicide attempters during the first period assessed by the study were single suicide attempters, while nine (64.29%) were multiple suicide attempters
Lopez-Morinigo et al. (2019) [26]	AESOP cohort, 34 (18.7%) subjects attempted suicide either before the first presentation or over the follow-up. Sixteen (8.8%) subjects had previous suicide attempts. Twenty-six (14.4%) subjects attempted to end their lives over the follow-up, including eight re-attempters (4.4%). Thirty-two (28.5%) GAP patients attempted suicide either before the first contact with services or over the study follow-up. Over the follow-up there were 18 (16.1%) suicide attempters and eight (7.1%) of these did so both before and after first contact with services, including three suicides. In total, over the follow-up periods, there were 44 suicide attempters and 80 suicidal events
Barret et al. (2010) [27]	Of the 194 patients, 89 (45.9%) were currently suicidal
Fedyszyn et al. (2011) [8]	Seventy-three (12%) attempted suicide during treatment. Of these 73, most (72.6%) attempted suicide on one occasion
Canal-Rivero et al. (2018) [28]	Fifty-one (9.9%) participants made at least one suicidal behavior. Thirty-six (70.59%) of these behaviors occurred during the first three years. Of those, seven died by suicide which reflects a proportionate suicide mortality of 46.7%
Ayesa-Arriola et al. (2018) [29]	Twenty-two (38% in the suicide behavior group) individuals with stable good insight attempted to end their lives during the follow-up, and 21 (37%) subjects with stable poor insight during the first two years, particularly within the first 200 days. When insight declined, five (9%) individuals attempted to end their lives between the second and the third year of follow-up, and when insight improved, three (5%) individuals made a suicide attempt. The six (10%) individuals who presented instability in the insight assessment showed suicidal behavior proximal to three-year follow-up
Pelizza et al. (2020) [30]	A 4.5% lifetime prevalence of suicide attempts was found in the FEP total group
Austad et al. (2015) [31]	Categorizing suicidal behavior into suicidal ideation (thoughts and plans) and suicide attempts revealed that our lifetime prevalence rates of suicidal ideation were around 40%. Prevalence of suicide attempts, 14.6% for lifetime and 6.9% for the past month

Demographic Predictors of Suicidal Behaviors in First-Episode Psychosis Patients

A total of 13 studies, encompassing 26,908 participants, were included in the analysis [8,20-31]. The mean age of each cohort is presented in Table 1. The risk of suicide was higher among adolescents in comparison to young adults [23]. Younger age at first presentation was found to be a positive predictor of suicidal behavior [26,31]. Furthermore, advancing age was associated with an increased risk of complete suicide [25]. The relationship between female gender and suicidal behavior was observed in both lifetime and current risk assessments [31]. Additionally, even after accounting for other risk factors, a longer duration of untreated psychosis (DUP) and younger age were linked to a higher prevalence of lifetime suicidal behavior [31].

Deliberate Self-Harm as a Risk Factor for Suicidal Behavior in First-Episode Psychosis Patients

Deliberate self-harm (DSH) encompasses intentional acts of non-fatal self-poisoning or self-injury, regardless of suicidal intent. This broad definition includes both non-suicidal self-injury, which involves self-harming behaviors without the intention to die, and suicide attempts [21]. Three studies reported self-harm as a predictor of suicidality in FEP patients [20-22]. In a retrospective analysis, around 11.1% of the total cohort experienced at least one DSH event, with the highest occurrence within the initial three months following the diagnosis of psychosis [21]. Moreover, a history of self-harm was the strongest predictor of future suicide attempts [21]. The likelihood of DSH increases over time, with adolescents facing a higher risk compared to young adults [21]. Notably, females and individuals with specific mental health disorders, such as ADHD, anxiety, disruptive behavior, and substance use, demonstrated an elevated risk of DSH [21]. Conversely, disabled or other eligibility types, non-Hispanic Black participants, and young adults, as opposed to adolescents, were associated with a decreased risk of DSH [21]. A study examining risk factors for DSH found that the suicide rates were significantly higher in their study cohort compared to the general population. The standardized mortality ratio (SMR) is an epidemiological measure comparing observed deaths in a specific population to the expected deaths in a standard population with similar age and sex distribution [32]. The SMR was 4.5 for the entire cohort, with adolescents having a higher SMR of 5.4, and young adults having an SMR of 3.2. The median follow-up time before suicide was longer for adolescents (501 days) compared to young adults (240 days) [21]. Those with a history of previous self-harm were approximately four times as likely to attempt suicide as those with no prior history [22].

Prevalence and Severity of Suicide Attempt in First-Episode Psychosis Patients

The number of suicide attempts was found to be positively correlated with the severity of symptoms of psychosis, as well as personality traits such as schizoid and sociopathic [22,25]. Theory of mind (ToM) is the cognitive ability to understand and represent the mental states of oneself and others, such as thoughts, beliefs, and intentions [33]. First-order false belief tasks involve inferring a false belief about the world, whereas second-order false belief tasks involve the ability to differentiate a false belief about another person’s beliefs [25]. In the ToM testing, patients with errors in false belief tasks were at high risk for multiple suicide attempts [25]. Additionally, multiple suicide attempters tend to make a first suicide attempt after FEP earlier than single suicide attempters [25]. Moreover, the presence of more severe symptoms of psychosis at the time of first contact with mental health services was associated with multiple suicide attempts [25]. In those with previous suicide attempts, depression increased the risk of suicidal behavior in early psychosis [22]. One of the statistically significant predictors of multiple SA was higher symptoms of depression at stabilization [22].

Role of Insight in Suicidal Behavior in First-Episode Psychosis Patients

The relationship between insight and suicidal behavior in patients with FEP remains unclear, with conflicting results from different studies. Some studies suggest that poor insight increases the risk of suicidal behavior over time, while changes in insight in both directions also increase the risk [26,27,29]. Another study suggested that depressive symptoms, higher insight, and beliefs about negative outcomes for psychosis are independently related to current suicidality; however, beliefs do not moderate the effect of insight on suicidality [27]. However, previous suicide attempts and depression appear to be associated with an increased risk of suicidal behavior in FEP patients, while no insight dimension is directly associated with an increased risk of suicidal behaviors [26]. Additionally, suicidal antecedents before the first presentation appear to influence the insight level [26].

Duration of Untreated Psychosis as a Predictor of Suicidality in First-Episode Psychosis Patients

DUP refers to the time between the onset of psychotic symptoms and the initiation of adequate treatment. There is growing evidence from these studies that a longer DUP is associated with poorer outcomes in patients with FEP and an increased likelihood of suicidal behaviors [24]. DUP was associated with a lifetime risk of suicidal behaviors [31]. Moreover, longer DUP was seen in male patients [31].

Psychiatric Comorbidities and Diagnoses of Psychosis in First-Episode Psychosis Patients

Austad et al. found a significant association between depressive symptoms and an increased risk of suicidal behavior in FEP patients, especially among females [31]. In contrast, males with FEP were more likely to be diagnosed with schizophrenia spectrum disorders compared to females [31]. Regarding diagnostic profiles, the majority of the study sample consisted of individuals with non-affective psychosis, while schizophrenia and schizophreniform disorder were prevalent diagnoses [8,29,30]. Comorbid conditions were common, including depression, substance abuse/dependence, anxiety disorders, and personality disorders [8]. Hallucinations and delusions at baseline were independent predictors of suicidal ideation over the study period [23].

Two studies emphasized the association between current depressive symptoms and schizophrenia spectrum disorders with current suicidality [26,27]. Suicidal patients demonstrated greater insight and held more pessimistic beliefs about psychosis, particularly regarding negative outcomes [27]. Previous suicide attempts and depression were identified as risk factors for suicidal behavior in early psychosis [26]. Furthermore, individuals diagnosed with depressive psychosis were nearly three times more likely to attempt suicide compared to those with schizophrenia/schizophreniform disorder [23].

*Other Predictors of Suicidal Behaviors in First-Episode Psychosis* *Patients*

Males with FEP exhibited poorer scores on the PANSS cognitive component and lower levels of functioning, but better scores on the PANSS depressive component [31]. A study focused on individuals with FEP examined the relationship between baseline Brief Psychiatric Rating Scale (BPRS) item four sub-scores and various factors. It found that younger FEP patients had higher scores on item 4, with positive correlations to PANSS sub-scores related to negative symptoms and general psychopathology. Patients aged 31-39, however, had lower item 4 scores compared to younger patients. PANSS sub-scores for guilt feelings and depression as strong predictors of suicidal ideation [30]. The BPRS is a widely used instrument for clinicians to assess the presence, severity, and 24 main psychiatric symptoms. The symptoms are rated on a scale ranging from 1 (not present) to 7 (extremely severe) [30].

Another study aimed to identify predictors of suicidal behavior during a three-year follow-up period [28]. Baseline assessments revealed that patients with suicidal behavior had higher scores on the CDSS and poorer premorbid adjustment [28]. Regarding cognitive function, patients with suicidal behavior performed worse in motor dexterity, working memory, and GCF. Additionally, a history of suicide attempts before FEP was more common in participants with suicidal behavior [28]. A binary regression model found that GCF and CDSS scores were significant predictors of suicidal behavior after FEP, with an accuracy of 91% in predicting the presence/absence of suicidal behavior [28]. The study further revealed that worse GCF was the most important baseline predictor of lifetime suicidality, a novel finding in FEP research [28]. This was especially true in patients diagnosed with major depressive disorder, wherein worse global neuropsychological functioning was associated with suicidal behavior [28].

Regarding completed suicide risk, older age, longer DUP, higher PANSS positive scores, and higher GAF symptomatology and disability scores were significant predictors [25]. Interaction effects among covariates were examined, and the final model showed that older age, longer DUP, and higher GAF disability scores increased the risk of suicide. Additionally, there was a significant interaction between PANSS positive and negative scores, indicating that an increase in the mean scores of these two factors increased the risk of suicide [25].

Discussion

The reviewed literature provides valuable insights into the relationship between FEP and suicidal behaviors in adolescents, transitional-age youth, and adults. DSH emerged as a significant risk factor for suicidal behavior in FEP patients [20-24]. The studies showed a high prevalence of DSH among adolescents, with rates as high as 11.1% during follow-ups [21]. Similarly, a study examining participants who were clinically at high risk for psychosis and individuals with FEP also found a high prevalence of suicidality and self-harm [34]. To add to these findings, a systematic review identified past or recent suicidal ideation, previous DSH, past depressive episodes, drug abuse or dependence, and a higher mean number of psychiatric admissions as risk factors for DSH in individuals with schizophrenia [35].

Insight, or the individual’s awareness and understanding of their illness, has been a subject of interest in relation to suicidal behavior in FEP. The findings in this area were somewhat conflicting, with some studies suggesting that poor insight increases the risk of suicidal behavior, while others did not find a direct association [27,29]. The findings from a community-based prospective cohort study provided further evidence that young adolescents with comorbid symptoms of depression and conduct disorder face an increased risk of experiencing suicidal ideation, recurrent suicidal ideation, and engaging in suicide attempts [36]. These results emphasize the importance of recognizing and addressing the mental health needs of this vulnerable population to prevent the occurrence of suicidal behaviors [36]. In particular, these individuals are more likely to have experienced suicidal thoughts compared to those without comorbidity [36]. This reiterates the importance of early identification and intervention for young adolescents with these comorbid symptoms to prevent the development of suicidal behaviors. These findings demonstrate the complex interplay between insight, depressive symptoms, and suicidal tendencies, underscoring the need for comprehensive assessments and tailored interventions addressing these factors, especially in adolescents and young adults.

The results of this review also highlight the importance of early intervention and reducing the DUP in mitigating suicide risk. There is evidence that longer DUP is associated with a higher risk of suicidal behavior in FEP patients [37]. This calls for early detection, timely access to mental health services, and effective treatment initiation for individuals experiencing their first episode of psychosis.

Regarding suicide timing, the review did not find a consensus; however, it is known that the highest suicide risk in adolescents with psychosis occurs very early in the disorder [38]. A significant percentage of individuals had attempted suicide before admission or during the first month of hospitalization [8]. In another study, most attempts occurred when patients were treated as outpatients and were in regular contact with the service [8]. These findings align with results from other studies on FEP, indicating that the risk is highest in the period preceding treatment initiation and during the first few months, even extending to the first year after onset [39,40].

Depression, distress with psychotic symptoms, fewer negative symptoms at first episode, positive symptoms, and anxiety disorders were identified to be associated in patients with FEP [22,26,27]. Depressive symptoms consistently showed a strong association with a higher risk of suicide attempts, particularly in adolescents with early psychotic disorders [17,22].

Although this review includes a broad range of age groups, it does identify the importance of examining the characteristics and timing of suicide attempts, especially in adolescents with psychotic disorders, to improve treatment and early detection, as well as to monitor and prevent attempts. Training primary care physicians in depression recognition and treatment, educating youths on depression and suicidal behavior, and conducting active outreach to psychiatric patients after discharge or a suicidal crisis is effective in preventing suicidal behavior [41]. During the first few years after the initial contact with mental health services, the risk of suicide increases significantly, underscoring the importance of early and intensive intervention measures. Implementing close patient engagement and vigilant symptom monitoring can effectively decrease suicide risk during this high-risk phase and positively influence long-term suicide risk. Specialized early intervention services play a vital role in delivering comprehensive treatment to young individuals facing their first episode of psychosis and their families, including thorough assessment and management of suicide risk [42].

Certain limitations were encountered in this review. A scarcity of studies specifically focused on adolescents with psychotic disorders and suicide attempts was noted. Consequently, our scope was broadened to include a wider age range to identify potential risk factors that may hold more significance in predicting suicide attempts in both adolescents and adults. However, not all studies included in our review provided stratified samples by age group, making it challenging to delineate all factors. Nevertheless, an increased risk in adolescents and young populations was indicated by the majority of studies.

To strengthen our findings and provide more definitive conclusions, further research is necessary. This includes conducting more extensive clinical follow-up studies, utilizing larger and more representative patient samples, as well as incorporating randomized controlled trials. By building predictive models, we can gain valuable insights into risk factors for suicide in patients with FEP, both in adolescence and adulthood.

Despite these limitations, the insights gathered from the reviewed studies contribute important knowledge to our understanding of suicide risk predictors in adolescent and adult patients with FEP. Given the aforementioned limitations, conducting additional studies with control groups and larger populations, including comprehensive descriptions of the population, symptoms, and treatments, would be valuable in definitively clarifying whether adolescents with psychotic disorders have a higher risk of suicide and/or suicide attempts, as well as in identifying specific risk profiles. There is a need for targeted interventions addressing self-harm behaviors, depressive symptoms, insight, and reducing the DUP. Future research should focus on further elucidating the mechanisms underlying the relationship between FEP and suicidal behaviors and developing effective preventive strategies to reduce suicide risk in this FEP population.

## Conclusions

This review examined various factors such as psychiatric, familial, and social factors, as well as previous self-harm, suicidal ideation, and comorbid mental health disorders. The findings suggest that depressive symptoms, substance use disorders, previous self-harm or suicidal ideation, and longer DUP are associated with an increased risk of suicidality. However, insight into psychosis and premorbid intellectual functioning did not show a direct association with suicidality. These findings serve to highlight the importance of early detection and intervention strategies targeting individuals at a heightened risk of suicide during the initial stages of psychosis.
